# Creating Coherence-Based Nurse Planning in the Perinatology Care System

**DOI:** 10.3390/healthcare10050925

**Published:** 2022-05-17

**Authors:** Windi Winasti, Sylvia G. Elkhuizen, Frits van Merode, Hubert Berden

**Affiliations:** 1 IQ Healthcare, Radboudumc, 6525 EP Nijmegen, The Netherlands; b.berden@etz.nl; 2 Erasmus School of Health Policy and Management, Erasmus University Rotterdam, 3062 PA Rotterdam, The Netherlands; elkhuizen@eshpm.eur.nl; 3 Care and Public Health Research Institute, Maastricht University, 6200 MD Maastricht, The Netherlands; f.vanmerode@maastrichtuniversity.nl; 4 Maastricht University Medical Centre+, 6229 HX Maastricht, The Netherlands; 5 Elisabeth-TweeSteden Ziekenhuis, 5022 GC Tilburg, The Netherlands

**Keywords:** nurses, flexibility, hospital care system, inpatient wards, capacity management

## Abstract

The combination of increasing demand and a shortage of nurses puts pressure on hospital care systems to use their current volume of resources more efficiently and effectively. This study focused on gaining insight into how nurses can be assigned to units in a perinatology care system to balance patient demand with the available nurses. Discrete event simulation was used to evaluate the what-if analysis of nurse flexibility strategies and care system configurations from a case study of the Perinatology Care System at Radboud University Medical Center in Nijmegen, the Netherlands. Decisions to exercise nurse flexibility strategies to solve supply–demand mismatches were made by considering the entire patient care trajectory perspective, as they necessitate a coherence perspective (i.e., taking the interdependency between departments into account). The study results showed that in the current care system configuration, where care is delivered in six independent units, implementing a nurse flexibility strategy based on skill requirements was the best solution, averaging two fewer under-/overstaffed nurses per shift in the care system. However, exercising flexibility below or above a certain limit did not substantially improve the performance of the system. To meet the actual demand in the studied setting (70 beds), the ideal range of flexibility was between 7% and 20% of scheduled nurses per shift. When the care system was configured differently (i.e., into two large departments or pooling units into one large department), supply–demand mismatches were also minimized without having to implement any of the three nurse flexibility strategies mentioned in this study. These results provide insights into the possible solutions that can be implemented to deal with nurse shortages, given that these shortages could potentially worsen in the coming years.

## 1. Background

The demand for hospital care has been growing in recent years due to an increase in the aging population [1] and developments in medical technology [2]. Given the increasing demand, the supply of hospital resources is expected to increase as well, leading to an adequate match between supply and demand. However, in reality, hospitals are experiencing a supply (e.g., nurse) shortage, and this could potentially worsen in the coming years [3,4].

The combination of increasing demand and a shortage of nurses puts pressure on the hospital care system to use the current volume of resources more efficiently and effectively [5]. The matching between the availability of resources on the one hand and, on the other, the demand for appropriate good-quality patient care is becoming the objective of resource planning and control in hospitals [5,6,7].

The matching between resources and demand in a specific department is also determined in part by other departments [8]. An example of this is the throughput problem concerning patients going from the intensive care departments to inpatient wards. A lack of a capacity limit on inpatient wards means that patients have to remain in the intensive care department longer than medically necessary, even though the cost of a stay in the intensive care department is higher than the cost of a stay in an inpatient ward. Inpatient wards, in turn, often have patients who are waiting for a place in post-hospitalization care, such as in a rehabilitation facility.

A recent study summarized ways of matching resources and demand in inpatient wards settings [9], such as by focusing on the outflows of patients; creating an extra capacity by discharging patients who are supposed to be discharged but who are still in the system due to non-practical reasons [10,11]; and focusing on capacity adjustments, i.e., creating extra capacity by reallocating the capacity from less-utilized wards to more highly utilized wards [5,12,13].

In this article, we focus on capacity adjustment, providing insights into how nurses can be scheduled and reallocated to units in a care system to balance patient demand with the number of available nurses. We achieve this by presenting four objectives: to provide insight into how decisions about the care system configuration, taken at the strategic level, influence under-/overstaffing occurrences at the real-time level; to report on which nurse flexibility strategy is best for minimizing under-/overstaffing occurrences; to report on the flexibility limit needed for minimizing under-/overstaffing occurrences; and how to create a reallocation process at the real-time level. A case study of the Perinatology Care System at the Radboud University Medical Center (Radboudumc) in Nijmegen, the Netherlands, is presented.

### 1.1. Nurse Capacity Planning and Control in Hospitals

Before a nurse actually cares for specific patients during a specific shift on a specific day, several stages of planning are required. As reported by earlier studies, capacity planning and control processes are hierarchically structured as being strategic, tactical, operational, and real-time (also referred to as operational online) [14,15]. Decisions made at an earlier level will govern the planning freedom at subsequent levels.

At the strategic level, hospitals decide how to organize the beds within the care system (e.g., how many beds a department should have, given the expected patient demand) and the process and product parameters necessary for providing care [15]. Based on this, hospitals subsequently decide whether nurses will be allocated and scheduled exclusively to decentralized departments or whether some of the nurses will be allocated to a centralized float pool [16,17]. At this level, hospitals also decide which methods will be used to deal with variability in patient arrivals. Several studies [18,19,20] have shown that when the number of resources (such as nurses) is flexible (i.e., the capability to increase or decrease the volume capacity), this can help meet fluctuations in patient demand in the departments. When nurses have a broad range of skills, this can also help to meet variable demand because it enables resources to flow where they are needed.

Once decisions have been made on how to organize beds and nurses, an aggregate plan is developed, still at the strategic level, to calculate the required number of full-time equivalent (FTE) nurses and the accompanying skill requirements per department. At the tactical level, the aggregate plan is then used to create a general rostering guideline (also referred to as a resource master plan) for each department, which, in turn, is used to create guidelines to determine the required number of nurses for every shift of the day. The output of this is a primary roster that a department’s planner can use when scheduling nurses on the duty roster.

At the operational level, individual nurses are then scheduled to work specific shifts 4 to 8 weeks in advance of the actual shifts. Patients being admitted for surgical procedures are also scheduled at this level, although the planning horizon for these patients is much shorter than that for nurses.

At the real-time level, due to the variability in both patient demand and the supply of resources during the actual working shift, the number of nurses planned for at the operational level may be insufficient to care for the actual number of patients. Conversely, the number of nurses planned for can also be too high and lead to overstaffing, which is a sign of inefficiency.

Recent studies have shown how mismatches between resources and patient demand can be alleviated if a resource can be reallocated during a short period of time that is closer to real-time to allow departments to adequately respond to variability in patient demand [13,21]. This “short-term” period can be defined as a minimum of a few hours to a maximum of one day, depending on the departments’ ability to mitigate capacity problems when understaffing occurs (e.g., by using on-call nurses, flexible nurses, nurses who are able to work overtime, and agency nurses). For example, Munavalli et al. [22] studied outpatient settings in which generic resources were rescheduled hourly within the shifts. For most inpatient wards, the reallocation of nurses is performed some hours before the start of each standard 8 h shift [23].

Because departments are interdependent, decisions to adjust or reallocate resources have to be made by considering the entire patient care trajectory perspective [8,22,24]. Munavalli et al. [21] suggested treating the hospital system as a closed-loop system. In a closed-loop system, capacity decisions are aimed at stability at a hospital-wide level rather than in a specific subsystem alone. Thus, this necessitates a coherence perspective [25] (i.e., taking the interdependency between departments into account) to solve supply–demand mismatches. When making capacity decisions, a coherence perspective is the demand and capacity status of all of the interdependent departments.

### 1.2. The Perinatology Care System at Radboudumc

Perinatology (also known as maternal–fetal medicine) is a rapidly growing field that is concerned with delivering care to mothers and newborns. A perinatology care system is often modeled after a complete hospital system, with pre- and post-care bed units and various levels of care units [26], but frequently on a smaller scale.

At Radboudumc (one of the eight academic hospitals in the Netherlands), the Perinatology Care System consists of two local systems (i.e., departments): Obstetrics and Neonatology. There are 70 beds in total. The Obstetrics Department has three units: the inpatient wards (25 beds for adults and 7 for newborns), the Obstetrics High Care (OHC) Unit (3 beds), and the delivery rooms (6 beds). These different units are based on the type of care provided to the adults (the mothers) and will hereafter be referred to as O1, O2, and O3, respectively. There are also three units in the Neonatology Department: the Neonatal Intensive Care Unit (NICU) (14 beds), the Neonatal Post-Intensive Care (Post-IC) Unit (4 beds), and the Neonatal High Care/Medium Care (HC/MC) Unit (11 beds). We will refer to these units as N1, N2, and N3, respectively. The nursing capacity is managed independently in each department, and these nurses do not move between departments. In other words, nurses are allocated to shifts in their dedicated unit within their dedicated department, either Neonatology or Obstetrics.

Although the care in the Neonatology Department and the Obstetrics Department is configured separately, in terms of patient demand, the Neonatology and Obstetrics Departments are interdependent. Each year, an average of 35% of newborns are transferred from the Obstetrics Department to the Neonatology Department. When the Neonatology Department is full, the Obstetrics Department refuses expectant mothers.

### 1.3. What This Article Contributes

In this article, we provide insight into how the planning and control of nurses in hospitals can be improved to minimize under-/overstaffing occurrences. Although recent studies have shown relevant results with regards to nurse planning and control in hospitals, to the best of the authors’ knowledge, most of them address one or two specific levels of planning. These include strategic decisions about the dimensioning of hospital departments [24,26,27,28], creating a resource master plan for tactical planning [29,30,31,32], allocating nurses to or scheduling nurses for a shift [16,33,34], and how to reallocate resources in a manner that is closer to real-time [21]. In this article, we report on the dynamic relationship between the decisions made at the strategic level and how these set the boundaries at the real-time level in hospital settings. We do this by providing insight into the consequences of care system configuration decisions made at the strategic level towards the under-/overstaffing occurrences at the real-time level. Additionally, for each type of care system configuration, we show which type of workforce flexibility is the best in terms of minimizing supply–demand mismatches.

Second, despite the growing interest in studying capacity management in perinatology care systems, we only found a limited number of published articles that address neonatology and obstetrics departments in a combined setting. Among these articles, obstetrics and neonatal unit settings are studied in Cochran and Bharti [26], Takagi et al. [35], Asaduzzaman et al. [27], and Ferraro et al. [36]. However, most of these studies dealt with capacity decisions at the strategic level. In this article, we provide insight into how nurses can be assigned to units in a perinatology care system to balance patient demand with the number of available nurses. In concrete terms, we answer the following questions:

- What is the optimal care system configuration for minimizing under-/overstaffing at the real-time level?
- What is the best nurse flexibility strategy for minimizing under-/overstaffing at the real-time level?
- What would the minimum limit of flexibility needed by the system to minimize under-/overstaffing be?
- How can nurses be reallocated to a shift to minimize under-/overstaffing?

## 2. Method

### 2.1. Defining Flexibility: The Skill Matrix and the Planning Horizon

At the strategic level, the care system configuration addresses the organization of beds and nurses in a care system. These decisions are about the number of beds per department and how nurses are allocated to the department based on the process and product parameters that are necessary for providing care. Given the decisions about the care system configuration, the skill matrix of the nurses and the patient categories within the care system is quantified. This skill matrix is necessary because it shows which types of nurses can work in each department. At the operational level, the planners from each department will schedule nurses based on the skill matrix. At the real-time level, such a skill matrix will indicate whether a nurse can be reallocated from one location to another. Since nurses are often only assigned to work in their dedicated department, this means that they are unable to move between departments.

To deliver a good standard of care for a given level of patient demand, departments must have sufficient resources (e.g., nurses). Unfortunately, many hospitals are experiencing a nurse shortage [3,37], making it challenging to deliver good-quality care. A study by Buchan and Aiken [3] showed that in hospitals with lower nursing staff levels, patient mortality was higher, and nurses were more likely to report burnout. One way to deal with the shortage of nurses and increasing (variable) demand is to create a nurse flexibility strategy.

We define flexibility as the capability of an organization to increase or decrease the resource capacity to meet variable demand [5,38,39]. A nurse flexibility strategy utilizes the fundamental components of flexibility: labor flexibility and volume flexibility. Labor flexibility means that nurses have a broad range of skills, so they are able to work in more than one department. Volume flexibility means that the number of nurses allocated to the departments depends on fluctuations in patient demand in the departments. There are many variants of nurse flexibility strategies in the nursing literature, such as “float pools”, “resource teams”, and “cross-trained nurses”, and these terms are used interchangeably [9]. For example, Gnanlet and Giland [18] studied in-house nurses who were trained to float between similar wards within a specialization. More reviews on nurse flexibility variants can be found in Dziuba-Ellis [39].

If managed properly, the nurse flexibility strategy can improve the quality of care and reduce staffing costs [12,39]. In terms of improving the quality of care, a nurse flexibility strategy can enable a unit to continue to operate, even when there are spikes in patient admissions or nurse absenteeism. Implementing such a strategy allows a unit to operate with the required number of nurses and, thus, reduces the risk of patient neglect and medication errors. Additionally, when nurses are properly trained to work in other units, they can supplement those working in short-staffed units to maintain quality [12].

**Figure 1:**
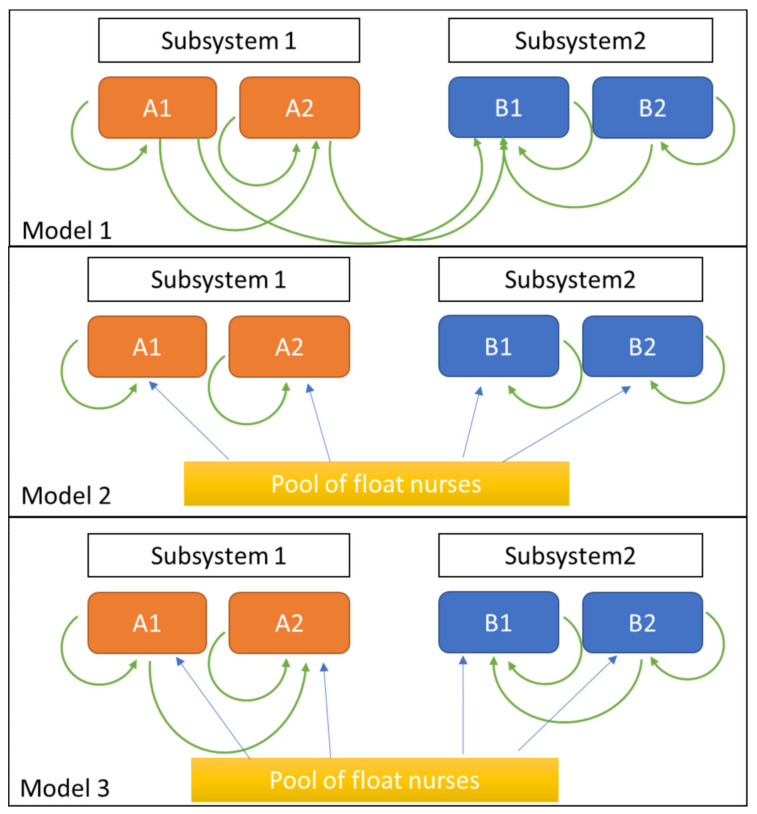
Nurse flexibility strategies, as explained in Section 2.2.

Given the chosen nurse flexibility strategy, a skill matrix is designed to show which nurses can work in other units and which nurses can float within the care system as a whole. In this article, we present three nurse flexibility strategy models, as shown in Figure 1.

To reallocate nurses at the real-time level, a planning horizon is needed. A planning horizon is defined as the interval needed to predict and subsequently reallocate nurses closer to the actual working shift. This is often based on the departments’ ability to react and solve capacity problems [23]. When the demand is too unpredictable, the planning of resources in a manner that is closer to real-time is needed [40]. In this study, the planning horizon is 8 h. This means that reallocation planning is performed after every 8 h of the actual working shift.

### 2.2. Nurse Flexibility Strategies

In this article, we present three nurse flexibility strategy models, which are shown in Figure 1: Model 1, a flexibility strategy based on skill requirements; Model 2, a flexibility strategy with a centralized float pool; and Model 3, a flexibility strategy that combines a centralized float pool with skill requirements.

### 2.3. Model 1: Nurse Flexibility Based on Skill Requirements

In Model 1, nurses from other units are reallocated based on skill requirements. This model is a modification of Inman’s [12] reciprocal pairs cross-training policy, whereby units pair up and each unit cross-trains one nurse to serve in the paired unit. In our study, we paired nurses based on their skill requirements. Specifically, nurses working in highly complex units are paired up with less complex units, allowing them to work in the paired (less complex) unit. Reallocation that is the other way around is not permitted. In the example in Figure 1, A1 and B2 are highly complex units. As such, nurses from A1 can care for patients in less complex units such as A2 and B1. Similarly, nurses from B2 can work in units B1 and A2. However, nurses from A2 and B1 cannot work in units A1 and B2.

### 2.4. Model 2: Nurse Flexibility with a Centralized Float Pool

Below, we model partial resource pooling, in which some nurses are dedicated to each unit, and the remaining nurses are pooled and shared between them. The number of nurses allocated to the float pool is decided based on the reallocation rate acquired from Model 1. For example, 38 nurses were scheduled to work during the next shift. Of the 38 nurses, 4 were supposed to be reallocated to meet the actual demand. The reallocation rate would, therefore, be 11% (i.e., 4 out of 38 nurses). We refer to this reallocation rate as the flex rate % (r).

Given that:

$$n=Shifts=\left\{day,\;evening,\;night\right\}\;$$

$$i=Units=\left\{1,2,3,\ldots \ldots ,k\;\right\}$$

let j be a location, then:

$$\;Z_{ji}=\mathrm{skill}\;\mathrm{matrix}=\left\{\begin{array}{c} 1,\;\;\;if\;nurses\;in\;location\;j\;can\;be\;reallocated\;to\;location\;i\\ 0,\;\;otherwise \end{array}\right.$$

$$\;N_{ji}^{n}=\;\mathrm{the}\;\mathrm{number}\;\mathrm{of}\;\mathrm{nurses}\;\mathrm{to}\;\mathrm{be}\;\mathrm{reallocated}\;\mathrm{from}\;\mathrm{location}\;j\;\mathrm{to}\;\mathrm{location}\;i\;in\;shift\;n;$$

$$M_{n}=\;\mathrm{the}\;\mathrm{total}\;\mathrm{number}\;\mathrm{of}\;\mathrm{reallocated}\;\mathrm{nurses}\;\mathrm{in}\;\mathrm{shift}\;n;$$

$$f_{n}=\mathrm{the}\;\mathrm{float}\;\mathrm{pool}\;\mathrm{in}\;\mathrm{shift}\;n$$

$$a_{ni}=\;\mathrm{the}\;\mathrm{number}\;\mathrm{of}\;\mathrm{available}\;\mathrm{nurses}\;\mathrm{in}\;\mathrm{shift}\;n,\;location\;i;$$

$$d_{ni}=\;\mathrm{the}\;\mathrm{number}\;\mathrm{of}\;\mathrm{required}\;\mathrm{nurses}\;\mathrm{for}\;\mathrm{shift}\;n,\;location\;i;$$

$$e_{ni}=\;\mathrm{the}\;\mathrm{excess}\;\mathrm{nurses}\;\mathrm{for}\;\mathrm{shift}\;n,\;location\;i;$$

$$e_{ni}=a_{ni}-d_{ni}\;\;\;\;\;\;\;\;\forall n,i;\;$$

$$e_{ni}=\left\{\begin{array}{c} <0,\;if\;location\;i\;is\;short\;of\;a\;nurse\;for\;shift\;n\;\\ =0,\;\;if\;location\;i\;has\;enough\;nurses\;for\;shift\;n\\ >0,\;if\;location\;i\;has\;more\;than\;enough\;nurses\;for\;shift\;n \end{array}\right..$$

We calculated the number of nurses that we want to reallocate:

(1) $$\;N_{ji}^{n}=\min\left(-e_{ni},e_{nj}\right)\times Z_{ji},\;\mathrm{where}\;e_{ni}\langle 0\;\mathrm{and}\;e_{nj}\rangle \;0\;\;\;\;\;\forall i,j,n\;\;i\;\neq \;j$$

The total number of reallocated nurses in shift *n* is calculated as

(2) $$M_{n}=\sum_{j}\sum_{i}N_{ji}^{n}\;\;\;\;\;\;\;\forall n$$

Therefore, the flex rate % (*r*) is

(3) $$r=\frac{\sum_{n}M_{n}}{\sum_{n}\sum_{i}a_{ni}}\times 100$$

The number of nurses in the float pool is

(4) $$f_{n}=\sum_{i}a_{ni}\times r\;\;\;\;\;\forall n.\;$$

After pooling the float nurses into the pool, the new number of available nurses during shift *n* and at location *i* ($a_{ni}^{f}$) is

(5) $$a_{ni}^{f}=a_{ni}-\left(a_{ni}\times r\right)$$

### 2.5. Model 3: Combination of Models 1 and 2

This model combines Models 1 and 2 in that the nurses are first reallocated from the centralized float pool, and this is then complemented by nurses from other units if extra nurses are still needed.

### 2.6. The Best Flexibility Strategy

The best strategy is the one that can minimize under-/overstaffing nurses per shift the most. For every Model *r*, where *r* = {1, 2, … *s*}, we calculated the average number of understaffed nurses per shift ($U_{r})$ and the average number of overstaffed nurses per shift ($O_{r}$). The best strategy, based on the average number of understaffed nurses ($B_{ru})$, can, therefore, be defined as $Max\;(U_{r}$), that is, the one that has the value closest to zero. Additionally, ($B_{ro})$ as $Min\;(O_{r},$), that is, the one with the lowest value of $O_{r}$, also has the value that is the closest to 0.

### 2.7. Reallocation Process at the Real-Time Level

The reallocation process involves reallocating nurses from the primary roster to a new roster, which can involve working in other units (within or outside the nurses’ dedicated departments), depending on the chosen flexibility strategy. We developed two fundamental steps for the reallocation process. Step 1 is a short-term demand prediction model that makes use of a coherence-based perspective. For Step 2, we developed three different nurse reallocation algorithms (which can be found in the Appendix A.2) based on the chosen flexibility strategy to meet the demand predicted in Step 1.

In Step 1, the short-term prediction is performed every 8 h using the units of the care system. For example, to predict the workload for the evening shift, the prediction process is started during the previous shift, the day shift. In our study, a Markov-based model was developed to predict demand in the care system. Since patient flows in a care system involve a number of states that evolve during the care process, these flows can also be represented by Markov models [4,41]. With Markov models, a matrix calculation is used to find the probability of patients moving from one unit to another in the future [42].

For Step 2, decisions to reallocate nurses are made simultaneously at the beginning of the planning horizon (i.e., every 8 h) for all units by taking into account the status (e.g., under-/overstaffed) of all of the units in the care system. In the literature, this process is referred to as centralized decision making [18]. The decisions to reallocate nurses should be made centrally by a central planner and governed by central rules, such as the reallocation algorithm that is provided in Appendix A.2.

### 2.8. The Optimal Care System Configuration

**Figure 2:**
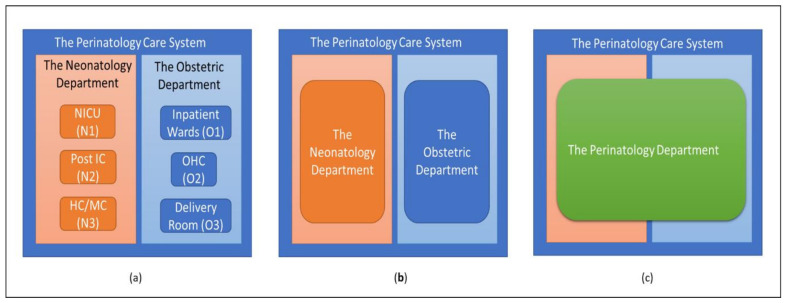
Care system configurations. Part (**a**): two departments in the current configuration, in which each department has three separate units and dedicated resources. Part (**b**): the resources of the three units in each department are pooled. Part (**c**): the care system consists of one large department (with its resources) to care for mothers and newborns.

In this study, we used the current care system configuration as the starting point for evaluating the three nurse flexibility models. As shown in Figure 2, part a, there are two departments in the current configuration, and each department has three units. We then experimented with the configurations shown in part b (Configuration 1) and part c (Configuration 2). In Figure 2, part b, the resources in the three units in each department are pooled. We then evaluated the responses of the three models (i.e., under-/overstaffing) accordingly. Finally, we experimented with the configuration in Figure 2, part c, in which the care system consists of one large department to care for mothers and newborns.

The optimal care system configuration is chosen based on several criteria, as we explain below.

1. The first criterion is the configuration that can minimize the average of under-/overstaffed nurses per shift, which are $B_{ru}$ and $B_{ro}$, with or without the use of a flexibility strategy.
2. The second criterion pertains to the regret that arises from having made a decision [43]. When a decision is made with the information that is currently available, it still might be wrong, given the uncertainty of several variables. This consequence of decision making under uncertain conditions is referred to as decision regret [43]. As explored in Bell [43], “regret” is quantified as the difference in the value between the real outcomes and the best outcomes produced by other alternatives. This definition allows there to be both positive and negative values of regret. In this study, we focused on the negative values of expected understaffing, given different demand conditions.
3. The third criterion is the configuration that requires the lowest training cost to implement. We define the cost of the training $\left(E_{r}\right)$ nurses need to acquire the skills required to be able to adopt the flexibility strategy in Model *r* (whereas r = {1, 2, … s}). If we refer to the model that requires the lowest cost as $\left(C_{r}\right)\;$=$\;Min\;\left(E_{r}\right)$, then the optimal configuration is defined as $B_{r}\cap C_{r}$. This configuration results in the minimum number of under-/overstaffed nurses per shift and is also the one that costs the least to implement.

### 2.9. Study Design

#### 2.9.1. Simulation and Optimization

**Figure 3:**
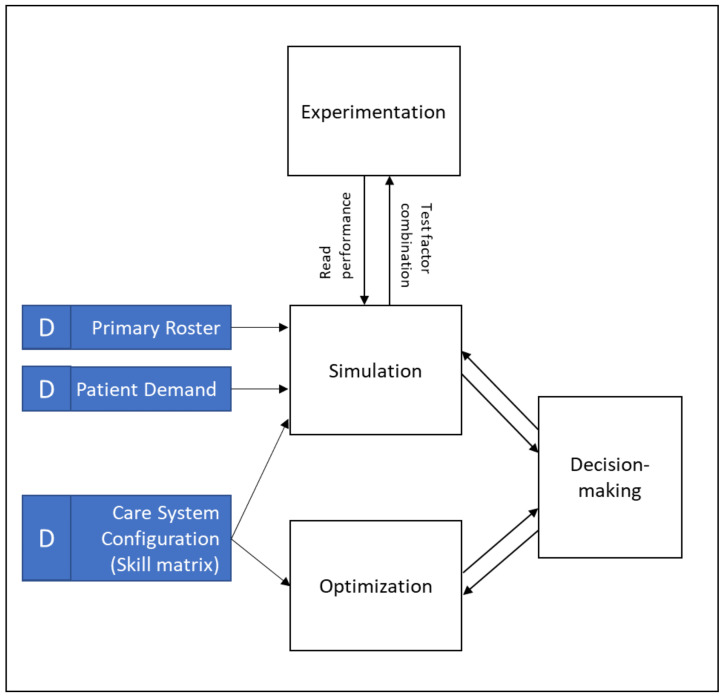
Study design.

As shown in Figure 3, the design of our study is based on the decision support system design published by van Merode et al. [44]. Given the initial care system configuration in the case study, we simulated the care delivery at the real-time level to measure the under-/overstaffing occurrences. We created a reallocation process to accommodate the flexibility strategy at the real-time level. The reallocation process was governed by the skill requirements that result from how the care system is configured and which flexibility strategy was chosen at the strategic level.

Given the skill requirements and the known primary roster (i.e., the available number of nurses planned for the shift), we experimented with two demand conditions: actual demand based on historical data (coefficient of variation (CV) of 31%) and uncertain demand (CV of 49%). The forward arrows between the processes in Figure 3 represent the flow of data to test interventions (i.e., the flexibility strategy), while the feedback arrows represent the flow of (simulation or experimentation) the outputs. The outputs were the average and the standard deviation of the under-/overstaffed nurses per shift. Based on the simulation outcome, we tried to optimize the care system configuration by adjusting the skill requirements (i.e., the forward arrow). Eventually, the feedback arrow will help provide decision makers with the following information:

- Which nurse flexibility strategy is the best for minimizing under-/overstaffing occurrences;
- How to reallocate nurses to a shift at the real-time level;
- Which care system configuration is optimal for the Perinatology Care System.

The details of the interventions and the accompanying variables can be found in Table 1.

#### 2.9.2. Data Analysis

To perform the short-term demand predictions (Step 1), we extracted several historical data (i.e., the year 2017) from the hospital information system. These data include patient movements between units, patient arrivals, length of stay (LOS), and the required nurse-to-patient ratio per unit. The prediction model was first developed in the spreadsheet program Excel (Microsoft Corporation, Redmond, WA, USA), and later on, MATLAB (The Math Works, Inc., Natick, MA, USA) was used to create the simulation scenarios.

For Step 2, the skill matrix was obtained through interviews with the head nurse of each department. We also obtained the number of nurses that can work in each unit. We modeled the skill matrix and the reallocation algorithm (for each flexibility strategy) in MATLAB. The output was the number of under-/overstaffed nurses per shift and unit. Furthermore, the output was analyzed in Excel to obtain the average and the standard deviation of the under-/overstaffed nurses in the care system and the related training costs.

## 3. Numerical Results

### 3.1. Data Analysis of the Perinatology Care System Radboudumc

As previously mentioned, there are 70 beds in total, as shown in Table 2 below. A total of 20% of the beds are beds that require highly specialized neonatology nurses, and 8% of the beds are beds that require highly specialized obstetric nurses. The rest of the beds require less specialization.

Below, Table 3 presents the size of the Perinatology Care System in terms of the number of nurses scheduled to work in a shift in each location. Each day, there are 28 nurses who are scheduled for the day shift, 21 nurses who are scheduled for the evening shift, and 17 nurses who are for the night shift. In total, there are 66 shifts per day. Given the number of available beds, the nurse-to-patient (NtP) ratio is calculated as provided in Table 4. More information about the theory and the calculation of the nurse-to-patient (NtP) ratio can be found in Elkhuizen et al. [45]. As for the current settings, the current nurse-to-patient (NtP) ratio is based on expert opinions and practical justifications, which have evolved from the past.

The number of nurses registered in each unit is provided in Table 5.

### 3.2. Reallocation Process at the Real-Time Level

#### 3.2.1. Step 1. Coherence-Based Demand

**Figure 4:**
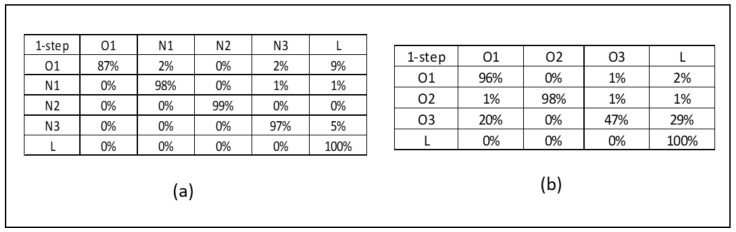
One-step transition matrix for Patient Group 1 (**a**) and Patient Group 2 (**b**).

The objective of Step 1 is to predict the need for an additional nurse during the upcoming shifts (i.e., 8 h) based on the interdependency between units. First, the transition matrix for each patient group is provided below. Given our analysis performed on the data extracted from the hospital information system, the P-matrix is the probability of patients moving from unit i to unit j during the next shift. For example, as shown in Figure 4a, there is a 4% chance of a newborn moving from unit O1 to N1 during the next shift. There is also a 4% chance they will move to N3, a 16% chance they will leave the system, and a 77% chance they will remain in O1. For the patient group comprising adults, the probability of patients moving from unit i to unit j during the upcoming shift(s) can be found in Figure 4b.

Using $C_{i}$ as the number of current patients in each unit i at the start of the day shift (n=0), which can be counted manually or acquired from the hospital information system, the transition matrix is used to calculate the total demand for nurses in unit *i* and in other units. Doing so takes the interdependency between units into account when predicting the demand for nurses.

**Figure 5:**
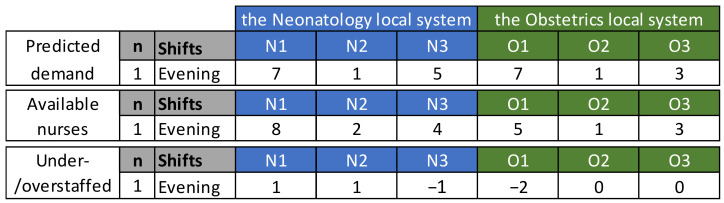
Example of predicting the need for extra nurses.

Next, we provide an example of how to quantify the need for an extra nurse. As shown below in Figure 5, based on the transition matrix and the number of current patients in each unit during the day shift, unit O1 needs seven nurses for the evening shift. Since there are only five scheduled (and available) nurses for the evening shifts, the final output for Step 1 is that unit O1 will need two extra nurses for the evening shifts and unit N3 will need one extra nurse for the evening shift.

#### 3.2.2. Step 2. Nurse Reallocation Policies

In this section, we present the results for Reallocation Models 1, 2, and 3 for under-/overstaffing occurrences, as provided in Figure 1.

Based on the interviews with the head nurses in both departments, we created the skill matrix shown in Table 6. For example, the most highly specialized nurses in the Neonatology Department are those working in N1. This means they can help out in N2 and N3 when needed because N2 requires less specialization than N1, and N3 requires the lowest level of specialization.

In the Obstetrics Department, O3 requires the highest level of nurse specialization, followed by O2, and finally, O1. In this case, the O3 nurses can help out in O2 and O1 when needed, but not the other way around. Furthermore, the nurses from N3 in the Neonatology Department can care for newborns in O1 in the Obstetrics Department. Nurses specialized in newborns in O1 in the Obstetrics Department can also help out in N3 in the Neonatology Department. We simulated Model 1 with the matrix shown in Table 6.

For Model 2 (i.e., R2), the size of the pool was calculated based on the prediction of the reallocation rate between nurses, as shown in Formula 3. We simulated Model 2 with the matrix presented in Table 7.

The best flexibility strategy is the one that can minimize under-/overstaffing occurrences the most. This is because understaffing can lead to refusals and delays, and overstaffing is a sign of cost inefficiency. As seen in Table 8, the average under-/overstaffing was generally the highest when there was no intervention (R0) in both demand conditions (e.g., CI = 31% and CI = 49%). For both demand conditions, when comparing the performance of the three models in the current care system configuration, it was determined that the ones that performed the best were Model 1 (R1) and Model 3 (R3) (i.e., both models obtained identical results). While Model 1 reallocates nurses based on skill requirements alone (i.e., highly specialized nurses can work in units requiring less specialized skills), Model 3 combines a centralized pool of float nurses, and reallocation is based on skill requirements.

Zooming in on the implementation of Model 2, the size of the float pool for R2 was 7%. In general, implementing Model 2 resulted in fewer under-/overstaffed nurses compared to no intervention at all. However, fewer improvements were gained than in Model 1. These results apply to all demand conditions.

**Figure 6:**
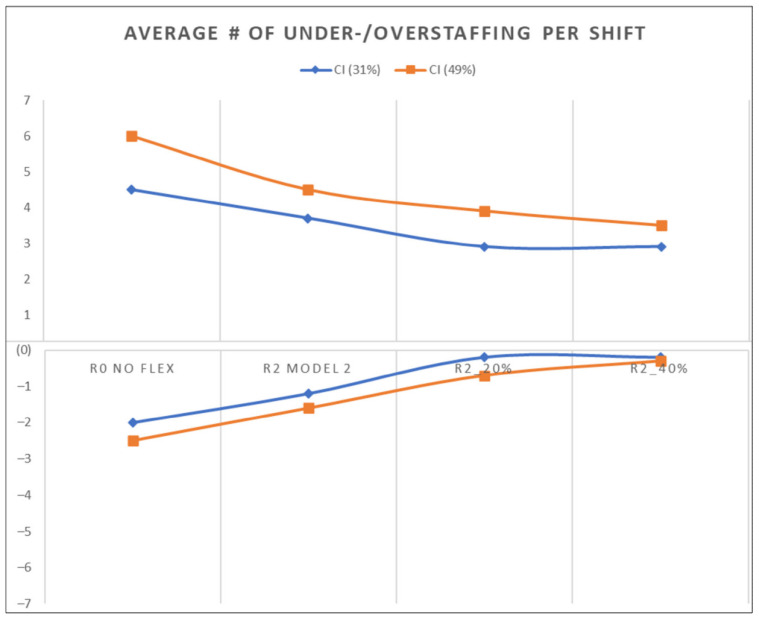
Results of the R2 modifications. The X-axis is the number of nurses, and the Y-axis is the different scenarios, including R0, R2, R2 with a 20% float pool, and R2 with a 40% float pool. The blue line represents the actual demand condition (CI:31%), while the red line represents highly variable demand (CI = 49%).

We then experimented with Model 2 for various float pool sizes (i.e., 20 and 40% of the total nurses in the system) and measured the effect towards the average number of under-/overstaffed nurses per shift. For both demand conditions, as the size of the float pool increased, the average number of under-/overstaffed nurses per shift shrank. As shown in Figure 6, when considering the actual demand condition, incrementing the size to 20% could minimize the average number of under-/overstaffed nurses per shift to an average of two fewer overstaffed nurses and two fewer understaffed nurses compared to R0 (i.e., no flexibility).

### 3.3. The Optimal Care System Configuration

As stated earlier, we experimented with different care system configurations. In Configuration 1, as depicted in Figure 2b, the units in each department were pooled to form a larger pool of resources. In Configuration 2, as shown in Figure 2c, all of the nurses in the Perinatology Care System can care for mothers and newborns.

In this section, we present the results of each configuration. We rounded each of the understaffed/overstaffed results. Rounding is carried out to obtain a value that is meaningful and relevant for practical purposes. We rounded our results to the nearest whole number. This means that for the average amount of understaffing per shift, any value between −0.5 and 0 has been rounded to 0, and any value between −0.5 and −1 has been rounded to −1.

The rounding value is particularly significant when dealing with understaffing requirements in practice. For example, in practice, the value of a 0.5 shift is equal to 4 care hours. When a department is understaffed by 2 or 3 care hours, the department’s patient care process will not be disrupted. These few hours can be dealt with informally, for example, by assigning a nurse who is currently working in non-patient care or on a study day to care for patients. However, if the understaffed value is more than 4 hours (i.e., half a shift), it is difficult for the department to deal with it in this way. In such cases, the department might need to make a formal request for a minimum of one extra shift (i.e., 8 care hours). The new results based on the rounding are presented in Table 9.

In terms of the training costs, implementing Model 1 involves no training costs for the current care configuration. For Model 2, 7% (based on the flex rate %) of the total number of nurses in the care system (176 nurses, as shown in Table 5) require training, which is a total of 12 nurses. If we define the cost of the training $\left(E_{r}\right)$ as EUR 20,000/nurse, Model 2 would cost EUR 240,000 to implement. As we increased the flex rate % to 20% and 40%, the cost increased to EUR 700,000 and EUR 1,400,000, respectively, as shown in Table 9.

In Configuration 1, all of the nurses in the Neonatology Department can care for patients in N1, N2, and N3. Similarly, all of the nurses in the Obstetrics Department can care for patients in O1, O2, and O3. The exception to this is that the nurses from N3 can also care for patients in unit O1 (newborns) in the Obstetrics Department. Likewise, the nurses from O1 can care for patients in N3 in the Neonatology Department. The updated skill matrix can be found in Appendix A.1, Table A1.

With Configuration 1, without any flexibility strategy (R4), the average number of under-/overstaffed nurses per shift is already minimized to an average of two fewer understaffed nurses and two fewer overstaffed nurses compared to R0. Experimenting with flexibility strategies R5, R6, and R7, the results are identical to those obtained for R4.

In terms of the training costs, to implement Configuration 1, the nurses from unit N2 (40 nurses) and N3 (14 nurses) need to be trained to care for all of the patients in the Neonatology Department. For the Obstetrics Department, most of the nurses are already trained to care for all of the patients in the Obstetrics Department. In our case study, only 10 nurses need training. Finally, Configuration 1 would cost EUR 1,280,000 (for a total of 64 nurses) to implement.

For Configuration 2, the updated skill matrix can be found in Appendix A.1, Table A2. For the results, the average number of under-/overstaffed nurses per shift was also identical to R4. In terms of the training costs, all of the nurses in the Neonatology Department should be trained to work in the Obstetrics Department and vice versa. In our case study, Configuration 2 would cost EUR 3,520,000 to implement.

#### 3.3.1. What Is the Optimal Care System Configuration?

As stated earlier, the optimal care system configuration is based not only on the best nurse flexibility strategy and the training costs for adopting the strategy but also on the minimum regret. As explained earlier, when a decision is made using the information that is currently available, it still might be wrong due to the uncertainty of several variables. The “regret” is quantified as the difference between the values of expected understaffing using the chosen strategy and the best outcomes produced by other strategies. In this study, we experimented with different demand conditions and evaluated the robustness of our chosen care system configuration. Responses from the three care system configurations are shown in Table 9.

Without implementing any of the nurse flexibility strategies mentioned in this study, the best configurations for minimizing under-/overstaffing occurrences were designing the Perinatology Care System as two large departments (i.e., Configuration 1 or R4) or one large department (i.e., Configuration 2 or R8). Otherwise, the current care system configuration achieves results similar to R4 and R8 with the nurse flexibility strategies presented in Models 1 and 3.

When analyzing the decision regret, when Model 1 or Model 3 were chosen as the strategies for the current demand condition (CI = 31%), our simulation showed that Models 1 and 3 were still the best strategies, even when we experimented with the system with the variable demand condition (CI = 49%).

In terms of the training costs for adopting the best strategy for minimizing under-/overstaffing occurrences, there are no training costs involved in implementing the flexibility seen in Model 1 (R1) with the current care system configuration. This is because in Model 1, the skills of the nurses in the highly specialized units allow them to work in units requiring less specialized skills. No additional training is needed for this. Without explicitly addressing the flexibility strategy provided in Figure 1, the care system should be configured as two large departments or one large department. The training costs would be EUR 1,280,000 and EUR 3,520,000, respectively.

#### 3.3.2. Reflection on the Size of Minimum Flexibility at the Current Perinatology Care System

Below, Table 7 presents the size of the Perinatology Care System in terms of the number of nurses scheduled to work during a shift at each location. Each day, there are 28 nurses who are scheduled for the day shift, 21 nurses who are scheduled for the evening shift, and 17 nurses who are scheduled for the night shift. In total, there are 66 shifts per day.

As indicated earlier, there are 66 shifts per day in total (see Table 3). Some of these nurses were pooled or reallocated to a new location to meet the variable demand. As shown in Figure 6 and Table 9, we showed that flexibility has a limit. Exercising flexibility (i.e., pooling nurses) below a certain limit will not substantially improve the performance of the system (i.e., R2). On the other hand, exercising flexibility above this limit is not efficient because it will not further improve the performance (i.e., R2_20%). For the actual demand in the studied setting (CI = 31%), the ideal limit is between 7% (i.e., the R2 flexibility rate) and 20% (i.e., the R2_20% flexibility rate).

- Size of Flexible Nurses in the Perinatology Care System

Specifically, as shown in Table 10, we present the empirical flexibility rate based on the ratio between the number of nurses reallocated to a new location during a shift and the total number of nurses scheduled to work during a shift. For the current setting, having a flexible rate of 13% is sufficient, as it covers 95% of the cases where there are two fewer overstaffed nurses and two fewer understaffed nurses compared to R0 (i.e., no flexibility).

When using a 13% flexibility rate, the studied care system needs four flexible nurses for the day shifts, three flexible nurses for the evening shifts, and two flexible nurses for the night shifts. The next question would be: “How do we assign nurses to these flexible shifts?”

**Figure 7:**
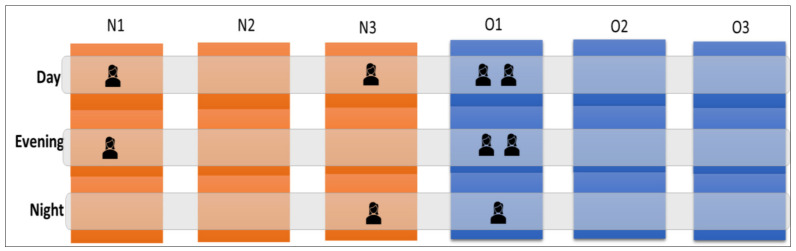
The configuration for flexible nurses.

One way to assign nurses to flexible shifts is by looking at the proportion of beds in a specific unit relative to the total number of beds in the care system. In other words, wards with more beds relative to the total number of beds in the system must assign more nurses to flexible shifts. Rationing the percentage provided in Table 2 with the required number of flexible shifts, we acquired the following configuration for flexible nurses (see Figure 7). In this example, because O1 has the biggest proportion of beds in the system, the unit contributed the most flexible nurses.

- Size of Flexible Beds in the Perinatology Care System

Based on the configuration of the skill mix for Model 1 (see Table 6), nurses can be reallocated to the other units. For example, N1 nurses can be assigned to the N2 and N3 units, and the N3 nurses can be assigned to the O1 units. This is determined by the assumption that the targeted units have beds available for patient admission. If all of the beds in the targeted units are fully occupied, in principle, patients can also be admitted to units with lower bed utilization as long as the skill matrix is met. Beds that can be used for these situations are further referred to as flexible beds.

**Figure 8:**
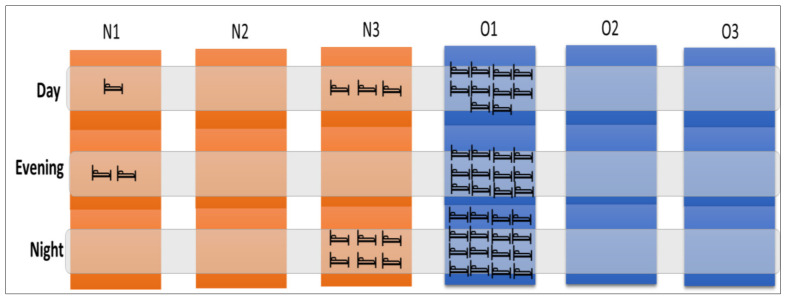
The configuration of flexible beds.

Based on the current nurse-to-patient ratio (see Table 4), the number of flexible beds in the Perinatology Care System is presented in Figure 8. In total, 14 flexible beds are needed for the day shifts, 14 flexible beds are needed for the evening shifts, and 22 flexible beds are needed for the night shifts. In the example using the current system, the beds in N3 can be used by newborns from O1 when needed and vice versa. Since nurses from N1 can care for patients in N2 and N3, patients in N2 or N3 can also be admitted to N1. However, this situation is less desirable because care delivery in N1 is more expensive than it is in N2 and N3.

## 4. Discussion

To deal with increasing (variable) demand and a limited supply of nurses, hospitals must use the current volume of nurses more efficiently and effectively. In this article, we provide insight into how nurses can be assigned to units in a perinatology care system to balance patient demand with the available nurses.

### 4.1. Which Nurse Flexibility Strategy Is Best?

Managing demand variability means that the variability will be buffered by capacity, time, or quality (or a combination of these) [5]. For example, a care system needs to design extra capacity (i.e., the capacity buffer) into the system when dealing with random patient demand (i.e., arrivals as well as acuity). Alternatively, they must accept the fact that patients will experience delays (i.e., the time buffer) in terms of entering the system or accept the risk of delivering poor-quality care (i.e., the quality buffer). Hopp and Lovejoy [5] argue that the size of the required buffer can be reduced by exploiting flexibility. As shown in several studies [18,19,20,46], when resources (such as nurses) are flexible, this can help to meet variable demand because it enables resources to flow where they are needed. Our findings align with the previous literature, which suggests the advantages of a nurse flexibility strategy when dealing with variable demand [12,18,39]. We showed that the number of under-/overstaffed nurses per shift decreased when any of the flexibility strategies investigated in this study were implemented.

Our findings also showed that the best policy is the one modeled by Model 1 (i.e., based solely on skill requirements, in which nurses from highly complex units can work in less complex units). Although the results from Model 3 (i.e., Model 1 with an additional centralized float pool) were identical to those of Model 1 in terms of the average number of under-/overstaffing occurrences per shift, our findings showed that an optimal condition had already been achieved by implementing Model 1. Complementing this model with a float pool strategy did not improve the system further. Even when we experimented with variable demand conditions, Model 1 remained the best strategy. Surprisingly, the results of the strategy modeled by Model 2 (i.e., with a centralized float pool) provided the lowest number of improvements compared to the other two strategies (those modeled by Models 1 and 3).

Why was Model 1 the best? Our findings suggest that flexibility has a limit. Pooling nurses below a certain limit might not improve the performance of the system, while pooling above this limit is not efficient. The float pool strategy (Model 2) can only succeed if the size of the capacity buffer is large enough to absorb the variable demand. This is because, during the actual working shift, the float pool size is fixed and cannot be increased or decreased when the level of short-term demand is different than what was predicted. Consequently, the float pool policy is highly dependent on the accuracy of the demand prediction.

On the other hand, Model 1 showed that the needed flexibility level varies between 7% to 20%, depending on the variable demand at the real-time level. This means that the number of these nurses can be increased or decreased (i.e., volume flexibility) depending on the demand status in the short term. For our Perinatology Care System, the number of scheduled nurses who work in highly specialized units is large enough to absorb the variable demand at the real-time level. Specifically, the volume of the capacity buffer embedded in Model 1 was larger than the number of dedicated nurses in the float pool (Model 2). For Model 2, the initial float pool size (i.e., the capacity buffer) was based on the prediction of the reallocation rate at the strategic level (i.e., a planning horizon of 12 months).

Consequently, our findings might be different if the care system only has highly (or less) specialized units with various specializations. In such a system, the capacity buffer should be created in another way. For example, another way to create the capacity buffer is to link units with similar skill requirements, such as nurses who work in a day surgery ward and nurses who work in a general surgery ward. Additionally, as suggested by Inman et al. [12], hospitals can link units by location proximity. Eventually, the criteria for enlarging the capacity buffer and, thus, enlarging the chain (i.e., which nurses/units are paired up with which nurses/units) depends on the practical considerations of the hospitals.

### 4.2. How Can Nurses Be Reallocated to a Shift to Minimize Under-/Overstaffing?

There are two steps to developing a reallocation process at the real-time level. First, a prediction process should be initiated to determine the short-term demand (such as during the next shifts, as shown in this study). By doing this, it will become apparent whether an extra nurse is needed in a specific unit. Based on the skill matrix (which is based on the chosen flexibility strategy at the strategic level or the care system configuration), decisions should be made to reallocate nurses to the unit where they are needed. The decision to reallocate nurses should be made centrally by a central planner and governed by central rules (e.g., the reallocation algorithm in Appendix A.2). The nurses can then be reallocated simultaneously by taking the statuses of all of the units in the care system into account [18]. By doing this, the planner will know which nurses will be working in which unit (or local system) in the care system.

### 4.3. Which Care System Configuration Is Optimal for the Perinatology Care System?

There is a connection between the decisions made at the strategic level (i.e., the care system configuration and the flexibility strategy) and how these set the boundaries at the real-time level in hospital settings. The combination of the care system configuration and the chosen flexibility strategy will result in the nurses’ skill matrix in each unit or department. It consequently dictates whether a nurse can be reallocated to other units at the real-time level. In our case study, care is delivered in six independent units. Because of this configuration, the skill requirements are different for each of the six units. To minimize the average number of under-/overstaffed nurses at the real-time level, the care system can opt for the flexibility strategy modeled by Model 1. Even when we experimented with variable demand, Model 1 was still the best, showing us that the strategy was robust against variable demand.

When perinatology care is organized into two large departments and skill requirements are tailored to these two departments (i.e., Configuration 1), this minimizes the average number of under-/overstaffed nurses at the real-time level without having to implement any flexibility strategy. Similarly, when the care is organized into one large department (i.e., Configuration 2), this also minimizes the average number of under-/overstaffed nurses per shift. However, one large department implies that all nurses are qualified to work throughout the department. To make this possible in the current situation, the hospital education department would have to provide extensive training for these nurses (e.g., 12 months for nurses in the Perinatology Care System), and there would be training costs involved.

An examination of the training costs for Model 1, the current care system configuration (R1), Configuration 1 (R4), and Configuration 2 (R8) shows that R1 involves the lowest training costs. We can regard R1 as a quick-win policy because the care system can implement this configuration immediately as long as the nurses are willing to be reallocated to other units when necessary.

### 4.4. What Are the Critical Success Factors for Applying the Proposed Method?

There are several practical factors to consider for the successful application of the proposed method. For example, we argue that the decision to reallocate nurses can be made centrally by a central planner and governed by central rules. This is aligned with a recent study by van der Ham et al. [47] in which they presented a social network analysis of hospitals making operational decisions (including the scheduling and rescheduling of resources). As can be observed in their study, decisional support by producing data and information for operational decision making seems to work best when provided to the operational decision makers compared to when it is provided to (tactical and strategic) manager(s). In our studied hospital, operational decision makers are the care professionals themselves and the operations staff, such as the planner and the head of nurses. Van der Ham et al. [47] argue that there is not much value in transferring these decisions to (tactical and strategic) managers, as they are often far from the focal point around which the decision making is taking place. On this note, the role of a central planner in a hospital will only become more important in minimizing supply–demand mismatches at the real-time operational level. Yet, it is also remarkable that very limited studies can be found on planners’ roles and qualifications for hospital capacity management.

There is funding associated with cross-training nurses. As discussed in van Merode et al. [40], care processes in hospitals vary in their uncertainty and stochastic characteristics. How much extra funding has to be found in order to implement cross-training will consequently depend on the type of care process. Our study found that if the patient demand for certain processes can be forecasted easily (i.e., given the lower coefficient of variation in this demand), fewer flexible nurses might be needed, and hence, fewer cross-training sessions would be required. Furthermore, if cross-trained nurses were paid a higher salary, cross-training costs would need to be weighed against the possibility and need to forecast demand for care processes.

Finally, our proposed method is designed for specific settings of the Perinatology Care System in Radboudumc. Our method would be applicable to other care systems with pre- and post-care bed units and various levels of care units (e.g., the cardiology care system with a first cardiac aid (FCA) unit (where emergency cardiac patients enter the hospital), the coronary care unit (CCU), and the cardiology inpatient unit (CIU)). However, variation would be needed when implementing it. In fact, the distinction between the approach as studied here and its implementation might not always be straightforward. As discussed in Vos et al. [48], with regard to how it is possible to standardize quality improvement methods and implement them in different departments, the distinction between design and implementation is fuzzy. Neglecting this might lead to failure. One of the essential elements for a successful implementation design is adequate information and communication technology (ICT) support [48]. Since our proposed method requires several data to work with, the role of decision support systems (DSS) as the ICT for nurse planning and control at the real-time planning level is only becoming more important.

## 5. Conclusions

### 5.1. Practical Implications

This article has shown how strategic decisions about the care system configuration can dictate how hospitals can meet the (variable) demand with a limited number of nurses. We have shown that if a care system is configured with several independent units, implementing a nurse flexibility strategy can help hospitals meet the (variable) demand with the current number of nurses. The best strategy was the reciprocal cross-pairing strategy, whereby units pair up and each unit cross-trains one nurse to serve in the paired unit. We showed an example of how highly specialized nurses can be paired up with units requiring lower care specialization (i.e., Model 1). Our simulation showed that this strategy performed better than the use of a centralized float pool. In alignment with what Jordan et al. [46] and Inman et al. [12] suggested, hospitals should always attempt to cross-pair as many units together as possible. Of course, it is important that practical considerations are also kept in mind. It is also important to encourage nurses to work in other units. As discussed by several researchers [49,50], nurses who regularly work in another unit may find it difficult to adjust to a new surroundings, new schedules, and new coworkers. To overcome these challenges, the head nurse can assign reallocations based on informed decisions about a nurse’s ability to help patients from the perspective of the care system. Informed decisions can be based on the expected demand, which is shared for all units in the care system. Such information may increase the sense of belonging of nurses in the two dedicated local systems of the care system.

Finally, our study contributes more insights to capacity management research in perinatology care systems. In our study, we provide examples where care delivery for mothers and newborns is modeled as a complete care system and how to deal with nurse shortages. With caution, the needed level of nurse flexibility in this study can be generalized by other perinatology care systems that also have similar characteristics to our settings, such as the ratio between the number of highly specialized beds and the normal beds, the skill mix characteristics between the units, and the demand characteristics of each unit.

### 5.2. Limitations and Future Research

The current study is not without limitations. First of all, as we mentioned earlier, we observed that when the Neonatology Department is fully occupied, the Obstetrics Department refuses expectant mothers. Mismatches between the resources and the patient demand that occurred in this study were potentially solved by adjusting the capacity of the system. However, we also observed that the Neonatology Department was sometimes fully occupied with babies waiting to be discharged from the unit (i.e., N1) instead of due to a lack of neonatology nurses. This indicates that the Perinatology Care System is also dealing with a lack of patient flow management, which involves organizing patient movements in a system instead of capacity management. One way to address patient flow management is to implement a discharge planning system, which ensures that patients who are medically ready to be discharged or transferred to other units (or hospitals) can be discharged on time [11]. Future research about this topic with regard to the Perinatology Care System is necessary.

Secondly, we started the prediction process during the day shift to predict the demand for care during the next shift (e.g., the next 8 h). Nurses are reallocated for the next shift. This means there is a response time of one shift, and this response time is needed to alleviate capacity problems. As this prediction process would also fit into a learning system, managers could identify the essential constraints and how to deal with them. The response time needed to alleviate capacity problems would then be expected to be shorter. Instead of performing the predictions for the next shift, managers could perform the prediction several hours ahead of every actual shift. In this way, resource planning would be based on actual (close to real-time) demand and could, therefore, potentially minimize occurrences of under-/overstaffing.

In addition, we simulated three flexibility models to evaluate which model has the best results in terms of minimizing under-/overstaffing occurrences. In practice, the results may be influenced by many other variables, such as nurse absenteeism and its effect on the primary roster. One way to incorporate nurse absenteeism is to update the primary roster at the start of the prediction process. As for other variables, future investigations are recommended. Nevertheless, while recognizing this limitation, we believe that our simulation models still point in the right direction in terms of results and will thus enable better-informed decisions about the effective use of nurses in hospitals.

Finally, in this study, our models provided the results as a real number. While looking for the optimal configuration, we rounded the number for practical purposes. Our approach differs from a mathematical optimization model, in which the feasible solution is based on the minimum (or maximum) value of all other feasible solutions without rounding. Thus, the optimal results determined using our approach might differ from the optimal results obtained when using one based on an optimization model, such as an integer programming model. However, in practice, our results provide more meaningful information for decision makers, particularly when dealing with understaffing requirements.

## Figures and Tables

**Table 1:** Study variables.

Care System Configuration	Controllable Variable (Name of Intervention)	Dependent Variable
Current configuration (6 separate units)	No flexibility intervention	R0
	Model 1	R1
	Model 2 Model 2 modifications in the size of the float pool to 20% and 40% of the total nurses in the system	R2 R2_20%, R2_40%
	Model 3	R3
Configuration 1 (2 departments)	No flexibility intervention	R4
	Model 1	R5
	Model 2	R6
	Model 3	R7
Configuration 2 (1 department)	No flexibility intervention	R8

**Table 2:** The number of beds in the Perinatology Care System.

Unit	N1	N2	N3	O1	O2	O3	Total
#Beds	14	4	11	32 (incl. 7 newborns beds)	3	6	70
% of total	20%	6%	16%	46%	4%	8%	100%

**Table 3:** The number of nurses scheduled per day.

Shifts	N1	N2	N3	O1	O2	O3	Total
Day	12	2	4	6	1	3	28
Night	8	2	2	5	1	3	21
Evening	7	2	2	2	1	3	17
Total	27	6	8	13	3	9	66

**Table 4:** The current nurse-to-patient ratio.

Shifts	N1	N2	N3	O1	O2	O3
Day	1:1	1:2	1:3	1:5	1:3	1:2
Evening	1:2	1:2	1:6	1:6	1:3	1:2
Night	1:2	1:2	1:6	1:16	1:3	1:2

**Table 5:** Number of available nurses in each unit.

Unit	Number of Nurses
N1	59
N2	40
N3	14
Total for the Neonatology Department	113
O1, O2, and O3	53
O1 and O2	5
O1	5
Total for the Obstetrics Department	63
Total for the Perinatology Care System	176

**Table 6:** Nurse matrix for Model 1 for the Perinatology Care System.

From/To	N1	N2	N3	O1 Newborns	O1 Adults	O2	O3
N1	1	1	1	0	0	0	0
N2	0	1	1	0	0	0	0
N3	0	0	1	1	0	0	0
O1 Newborns	0	0	1	1	1	0	0
O1 Adults	0	0	0	1	1	0	0
O2	0	0	0	1	1	1	0
O3	0	0	0	1	1	1	1

**Table 7:** Nurse matrix for Models 2 and 3 for the Perinatology Care System.

From/To	N1	N2	N3	O1 Newborns	O1 Adults	O2	O3
N1	1	1	1	0	0	0	0
N2	0	1	1	0	0	0	0
N3	0	0	1	1	0	0	0
O1 Newborns	0	0	1	1	1	0	0
O1 Adults	0	0	0	1	1	0	0
O2	0	0	0	1	1	1	0
O3	0	0	0	1	1	1	1
Float Pool	1	1	1	1	1	1	1

**Table 8:** Results of simulation with the current care system configurations using the actual demand and variable demand.

Configuration	Response	Actual Demand (CI = 31%)		Variable Demand (CI = 49%)	
		Average Understaffed (±SD)	Average Overstaffed (±SD)	Average Understaffed (±SD)	Average Overstaffed (±SD)
Current Configuration	R0 No flex	−1.6 (0.7)	4.5 (1.9)	−2.4 (1.1)	5.7 (2.4)
	R1 Model 1	−0.3 (0.6)	2.8 (2.1)	−0.5 (0.9)	3.5 (2.6)
	R2 Model 2	−1.2 (1.1)	3.7 (1.8)	−1.6 (1.4)	4.5 (2.4)
	R3 Model 3	−0.3 (0.6)	2.8 (2.1)	−0.5 (0.9)	3.5 (2.6)

**Table 9:** Results for different configurations.

Configuration	Response	Average Understaffed (Rounded)	Average Overstaffed (Rounded)	Cost of Training
Current Configuration (a)	R0 No flex	−2.0	5.0	EUR 0
	R1 Model 1	0.0	3.0	EUR 0
	R2 Model 2	−1.0	4.0	EUR 240,000
	R2_20%	0.0	3.0	EUR 700,000
	R2_40%	0.0	3.0	EUR 1,400,000
	R3 Model 3	0.0	3.0	EUR 240,000
	R0 No flex_Variable demand	−2.0	6.0	EUR 0
	R1 Model 1_Variable demand	−1.0	4.0	EUR 0
	R2 Model 2_Variable demand	−2.0	5.0	EUR 240,000
	R2_20%_Variable demand	−1.0	4.0	EUR 700,000
	R2_40%_Variable demand	0.0	4.0	EUR 1,400,000
	R3 Model 3_Variable demand	−1.0	4.0	EUR 240,000
Configuration 1 (b)	R4 No flex	0.0	3.0	EUR 1,280,000
	R5 Model 1	0.0	3.0	EUR 1,280,000
	R6 Model 2	0.0	3.0	EUR 1,280,000
	R7 Model 3	0.0	3.0	EUR 1,280,000
Configuration 2 (c)	R8 No flex	0.0	3.0	EUR 3,520,000

**Table 10:** The empirical results of flexibility rate from simulating Model 1.

Actual Demand (CI = 31%)		
Flexibility Rate %	Frequency	Cumulative %
0	3	0.82%
2	8	3.01%
4	20	8.49%
5	96	34.79%
7	71	54.25%
9	114	85.48%
11	26	92.60%
13	12	95.89%
15	7	97.81%
16	5	99.18%
18	3	100.00%
More	0	100.00%

## Appendix A

### Appendix A.1. The Updated Skill Matrix

**Table A1:** Skill matrix for Model 1 for the Perinatology Care System, Configuration 1.

From/To	N1	N2	N3	O1 Newborns	O1 Adults	O2	O3
N1	1	1	1	0	0	0	0
N2	1	1	1	0	0	0	0
N3	1	1	1	1	0	0	0
O1 Newborns	0	0	1	1	1	1	1
O1 Adults	0	0	0	1	1	1	1
O2	0	0	0	1	1	1	1
O3	0	0	0	1	1	1	1

**Table A2:** Skill matrix for Models 2 and 3 for the Perinatology Care System, Configuration 1.

From/To	N1	N2	N3	O1 Newborns	O1 Adults	O2	O3
N1	1	1	1	0	0	0	0
N2	1	1	1	0	0	0	0
N3	1	1	1	0	0	0	0
O1 Newborns	0	0	0	1	1	1	1
O1 Adults	0	0	0	1	1	1	1
O2	0	0	0	1	1	1	1
O3	0	0	0	1	1	1	1
Float Pool	1	1	1	1	1	1	1

### Appendix A.2. The Reallocation Algorithm Based on the Chosen Flexibility Strategy

*Notation*:

$$\begin{array}{c} \;n=Shifts=\left\{day,\;evening,\;night\right\}=\left\{1,2,3\right\}\;; \end{array}$$

$$\begin{array}{c} \;A=Location\;in\;Local\;system\;1=\left\{N1,\;N2,\;N3,\;O1S1\right\}; \end{array}$$

$$\begin{array}{c} \;B=Location\;in\;Local\;system\;2\;\left\{O1S2,\;O2,\;O3\right\}; \end{array}$$

$$\begin{array}{c} \;i=All\;units\;location=A\cup B=\left\{N1,\;N2,\;N3,\;O1S1,\;O1S2,\;O2,\;O3\right\}. \end{array}$$

*Let*j*be a location, then*:

$$\begin{array}{c} \;Z_{ji}=skill\;matrix=\left\{\begin{array}{c} 1,\;\;\;if\;nurses\;in\;location\;j\;can\;be\;reallocated\;to\;location\;i\\ 0,\;\;otherwise \end{array}\right.\; \end{array}$$

$$\begin{array}{c} \;a_{ni}=the\;number\;of\;available\;nurses\;for\;shift\;n,\;location\;i\;; \end{array}$$

$$\begin{array}{c} \;d_{ni}=the\;number\;of\;required\;nurses\;for\;shift\;n,\;location\;i; \end{array}$$

$$\begin{array}{c} \;e_{ni}=the\;excess\;nurses\;for\;shift\;n,\;location\;i; \end{array}$$

$$\begin{array}{c} \;e_{ni}=a_{ni}-d_{ni}\;\;\;\;\;\;\;\;\forall n,i; \end{array}$$

$$\begin{array}{c} \;e_{ni}=\left\{\begin{array}{c} <0,\;if\;location\;i\;is\;short\;of\;a\;nurse\;for\;shift\;n\;\\ =0,\;\;if\;location\;i\;has\;enough\;nurses\;for\;shift\;n\\ >0,\;if\;location\;i\;has\;more\;than\;enough\;nurses\;for\;shift\;n \end{array}\right.; \end{array}$$

$$\begin{array}{c} \;N_{ji}^{n}=the\;number\;of\;nurses\;to\;be\;reallocated\;from\;location\;j\;to\;location\;i\;in\;shift\;n; \end{array}$$

$$\begin{array}{c} \;M_{n}=the\;total\;number\;of\;reallocated\;nurses\;in\;shift\;n. \end{array}$$

$$M_{n}=\sum_{j}\sum_{i}N_{ji}^{n}\;\;\;\;\;\;\;\forall n\;$$

- Model 1: Prioritization-Based Nurse Reallocation

Step 1: Let n be a shift. For each location *i*, calculate the excess nurses, $e_{ni}$.

Let

$$E_{n}=\left\{e_{nO1S1,\;}e_{nN3,\;}e_{nN2,\;}e_{nN1,\;}e_{nO1S2,\;}e_{nO2,\;}e_{nO3}\right\}$$

Step 2: According to the following order, $a=\left\{O1S1,\;N3,\;N2,\;N1\;\right\}$ and $b=\left\{O1S2,\;O2,\;O3\right\}$, fulfill the nurse requirements. Suppose *i* is a location in one of the local systems. Find the first location *j* in E with $e_{nj}>0$ and j≠i.

Condition 2a: First, calculate the number of nurses to be reallocated to location *i* within the same local system.

$$N_{ji}^{n}=\min\left(-e_{ni},e_{nj}\right)\times Z_{ji}$$

where $e_{ni}\langle 0\;\mathrm{and}\;e_{nj}\rangle \;0,\;\mathrm{and}\;i\;\mathrm{and}\;j\;\mathrm{are}\;\mathrm{in}\;\mathrm{the}\;\mathrm{same}\;\mathrm{local}\;\mathrm{system}$.

Condition 2b: If there are no nurses to be reallocated to location *i* within the same local system, then look in another local system.

$$N_{ji}^{n}=\min\left(-e_{ni},e_{nj}\right)\times Z_{ji}$$

where $e_{ni}\langle 0\;\mathrm{and}\;e_{nj}\rangle 0,\;\mathrm{and}\;i\;\mathrm{and}\;j\;\mathrm{are}\;\mathrm{in}\;\mathrm{the}\;\mathrm{different}\;\mathrm{local}\;\mathrm{system}$.

Condition 2c:

$$N_{ji}^{n}=0$$

If

$$e_{ni}\geq 0,\;\;\mathrm{all}\;\mathrm{other}\;e_{nj}\leq 0\;\;\;\;\forall j\neq i\;$$

Step 3: Update the excess nurses, $e_{ni},$ and the nurse schedule, $a_{ni}$, accordingly.

$$e_{ni}=e_{ni}+N_{ji}^{n}$$

$$a_{ni}=a_{ni}+N_{ji}^{n}$$

$$e_{nj}=e_{nj}-N_{ji}^{n}$$

$$a_{nj}=a_{nj}-N_{ji}^{n}$$

If $e_{ni}<0$, repeat Step 2 to fulfill the requirements from the next location j; otherwise, go to Step 4.

Step 4: When all of the locations in shift *n* have been checked and the necessary reallocations have been made, repeat Steps 1 to 4 with *n* =n+1.

- Model 2: Cross-Trained Float Nurses

Step 1: Let *n* be a shift; for each location *i,* calculate the number of nurses to be reallocated during a particular shift *n* to a particular location *i* $\left(N_{i}^{n}\right).$

$$N_{i}^{n}=\left\{\begin{array}{c} 0,\;\;if\;e_{ni}>0\;or\;f_{n}=0\\ \min\left(f_{n},e_{ni}\right),\;\;\;if\;e_{ni}<0\;and\;f_{n}>0 \end{array}\right.$$

Step 2: Update the number of excess nurses $\left(e_{ni}\right)$ for every shift *n* and location *i*.

$$e_{ni}=e_{ni}+N_{i}^{n}\;$$

Step 3: Update the number of flexible nurses in the float pool $\left(f_{n}\right)$.

$$f_{n}=f_{n}-N_{i}^{n}$$

Step 4: Update the nurse schedule $(a_{ni}^{f}$), given the updated number of nurses in the float pool $\left(f_{n}\right)$.

$$a_{ni}^{f}=a_{ni}^{f}+N_{i}^{n}$$

Step 5: Repeat Steps 1 to 4 with *n* =n+1.

- Model 3: Cross-Trained Float Nurses and Nurse Reallocation with Priority

Step 1: Let *n* be a shift for each location *i* and calculate the excess nurses, $e_{ni}$.

Let

$$E_{n}=\left\{e_{nO1S1,\;}e_{nN3,\;}e_{nN2,\;}e_{nN1,\;}e_{nO1S2,\;}e_{nO2,\;}e_{nO3}\right\}a=1,$$

Step 2: Let $N_{i}^{n}$ be the number of nurses reallocated to location *i* during shift *n*.

Condition 2a: First, check the flexible nurses.

$$N_{i}^{n}=\min\left(-e_{ni},f_{n}\right)\;if\;f_{n}>0$$

where $e_{ni}<0$, in other words, location *i* is short of nurses.

Condition 2b: If $f_{n}=0$, then look for nurses within the same local system.

Suppose i is a location in one of the local systems, and *j* ≠i is the first location with $e_{nj}>0$ in $E_{n}$, then nurses can only be found if j and *i* are either both in *a* or both in b.

$$N_{i}^{n}=\min\left(-e_{ni},e_{nj}\right)\times Z_{ji}\;if\;f_{n}=0,e_{ni}<0,\;e_{nj}>0$$

Condition 2c: If $f_{n}=0$ and if there are no nurses to be reallocated to location *i* within the same local system, then look in another local system.

Thus, the conditions i∧ j∈a and i∧ j∈b are relaxed, and *j* ≠i is the first location in $E_{n}$ for which $e_{nj}>0$.

$$N_{i}^{n}=\min\left(-e_{ni},e_{nj}\right)\times Z_{ji}\;if\;f_{n}=0,e_{ni}<0,\;e_{nj}>0$$

Step 3: Update the excess nurses, $e_{ni},$ and the nurse schedule, $a_{ni}^{f}$, accordingly.

$$e_{ni}=e_{ni}+N_{i}^{n}$$

$$a_{ni}^{f}=a_{ni}^{f}+N_{i}^{n}$$

$$e_{nj}=e_{nj}-N_{i}^{n}$$

$$a_{nj}^{f}=a_{nj}^{f}+N_{i}^{n}$$

If $e_{ni}<0$, repeat Step 2; otherwise, go to Step 4.

Step 4: When all of the locations in shift *n* have been checked and the necessary reallocations have been made, repeat Steps 1 to 4 with n=n+1.
